# Pupillary Pain Index-Guided Postoperative Pain Therapy in ENT Surgery: A Randomized Trial

**DOI:** 10.3390/jcm15020462

**Published:** 2026-01-07

**Authors:** Marita Windpassinger, Michal Prusak, Lusine Yeghiazaryan, Robin Ristl, Sascha Ott, Lukas M. Müller-Wirtz, Kurt Ruetzler

**Affiliations:** 1 Department of Anesthesia, Intensive Care Medicine and Pain Medicine, Division of Pain Medicine, Medical University of Vienna, 1090 Vienna, Austria; 2 Outcomes Research Consortium, Houston, TX 77030, USA; sascha.ott@dhzc-charite.de (S.O.); lukas.mueller-wirtz@uk-erlangen.de (L.M.M.-W.);; 3 Karl Landsteiner Institute for Perioperative Medicine, 4020 Linz, Austria; 4 Department of Anesthesia, Intensive Care Medicine and Pain Medicine, Division of General Anaesthesia and Intensive Care Medicine, Medical University of Vienna, 1090 Vienna, Austria; michal.prusak@meduniwien.ac.at; 5 Center for Medical Data Science, Institute of Medical Statistics, Medical University of Vienna, 1090 Vienna, Austria; lusine.yelusine.yeghiazaryan@meduniwien.ac.at (L.Y.); robin.ristl@meduniwien.ac.at (R.R.); 6 Department of Cardiac Anaesthesiology and Intensive Care Medicine, Deutsches Herzzentrum der Charité, 13353 Berlin, Germany; 7 Department of Anesthesiology, Friedrich-Alexander-Universität Erlangen-Nürnberg, Universitätsklinikum Erlangen, 91054 Erlangen, Germany; 8 Department of Anesthesiology and Intensive Care Medicine, Ordensklinikum Linz, 4020 Linz, Austria

**Keywords:** anesthesia, pupillometry, pupillary pain index, nociception, postoperative pain, postoperative opioid consumption, pain therapy

## Abstract

**Background:** Postoperative pain levels and opioid requirements vary considerably, even among patients undergoing similar surgical interventions. The pupillary pain index—a pupillometry-derived measure of nociception-antinociception balance—may help individualize postoperative analgesia. We therefore tested the hypothesis that a pupillary pain index-guided opioid titration at the end of surgery reduces postoperative pain and opioid consumption compared with standard care. **Methods:** At the end of surgery, a portable infrared pupilometer was used to measure pupillary dilation reflex during stepwise tetanic stimulation (10–60 mA), generating a pupillary pain index score. Adult patients undergoing elective ear-nose-throat surgery under general anesthesia were randomized to pupillary pain index-guided opioid therapy or standard care. Opioid administration in the pupillary pain index group followed predefined pupillary pain index cutoffs; in the control group, analgesia was managed per routine practice. Postoperative opioid consumption and pain—assessed using a numerical rating scale (NRS, 0–10)—were recorded every 30 min for 2 h in the post-anesthesia care unit. Linear models with covariates including remifentanil, weight, nose surgery, and sex were calculated to compare outcomes between groups. **Results:** Mean (±SD) opioid consumption during the first 2 postoperative hours was 4.9 ± 4.3 mg in the pupillary pain index-guided group and 6.5 ± 4.3 mg in the control group (adjusted *p* = 0.12). Mean pain scores were 2.0 ± 1.1 and 2.6 ± 1.4, respectively (adjusted *p* = 0.10). **Conclusions:** Pupillary pain index-guided analgesia resulted in a nearly 25% reduction in opioid consumption and lower pain scores, although not statistically significant. This suggests that PPI-guided analgesia is not inferior to standard care in terms of pain management.

## 1. Introduction

Despite growing focus on individualized postoperative pain management, many patients continue to experience moderate-to-severe postsurgical pain. Postoperative pain is among the most relevant perioperative concerns for patients during the perioperative period and is a key factor influencing overall recovery [1,2]. Pain perception is inherently subjective, and patient-reported pain scores often correlate poorly with observable behavior. This is a limitation of commonly used tools such as the visual analog scale (VAS) or numeric rating scale (NRS) [1]. This challenge is pronounced in specific patient populations, including elderly patients with cognitive impairment, children, patients recovering from anesthesia, and patients undergoing procedures that may temporarily impair speech, such as ear–nose–throat (ENT) surgery. These factors increase the risk of both over- and under-treatment with opioids.

Although subjective pain scores remain the clinical standard, objective nociception monitoring may enable more precise titration of analgesics, potentially reducing opioid exposure and its associated side effects. However, many patients still wake up with moderate-to-severe pain [2], likely because clinicians primarily rely on relatively insensitive autonomic responses such as cardiovascular reactivity or motor responses as indicators of pain and nociception. Several technology-based tools have been developed to address this gap, including the Analgesia Nociception Index (ANI), Surgical Pleth Index (SPI), Nociception Level Index (NoL), and pupillometry. ANI relies on heart rate variability, SPI on photoplethysmographic and cardiovascular parameters, and NoL integrates multiple physiological signals, including photoplethysmography and skin conductance, into a machine-learning–based composite score. In contrast, the pupillary pain index (PPI) is derived from the pupil dilation reflex, providing a standardized nociceptive stimulus that is closely linked to central opioid effects [3,4,5,6,7,8,9].

Pupillometry offers a practical approach by quantifying the pupillary dilation reflex (PDR), which reflects the central opioid effect. This method can be used to evaluate analgesic efficacy and ultimately optimize opioid dosage titration [10,11,12,13]. The magnitude of the PDR in response to an evoked stimulus (wound pressure) rather than pupil size alone reflects the nociceptive-antinociceptive balance and has been shown to relate to postoperative verbal pain scores and opioid requirements [1,14,15]. In pediatric cohorts, pupillary metrics, including maximum pupil size, variation, and maximum constriction velocity, correlate with pain scores [16,17].

By utilizing the PDR, the pupillary pain index (PPI) score can be generated through incremental electrical stimulation (10–60 mA) with a portable pupilometer [14]. This technique requires unconsciousness, making it suitable for intraoperative assessment. Previous trials demonstrated that PPI-guided remifentanil titration during surgery reduced both postoperative pain and analgesic consumption for up to 12 h [15,16,17,18].

Therefore, we tested whether optimizing the intraoperative nociception–antinociception balance through PPI-guided opioid titration administered just before emergence from general anesthesia would reduce both postoperative opioid consumption and early postoperative pain, as reported using NRS scores in the recovery room.

## 2. Materials and Methods

This randomized trial was approved by the Ethics Committee of the Medical University of Vienna (approval 2112/2019; 25 April 2020). All participants provided written informed consent. The trial was prospectively registered at ClinicalTrials.gov (NCT04176289; 22 November 2019) prior to enrollment.

### 2.1. Patients

Adults aged ≥18 years, classified as American Society of Anesthesiologists (ASA) physical status I-II, who were scheduled to undergo elective ENT surgery at the Department of Otorhinolaryngology at the Medical University Hospital Vienna, 1090 Vienna, Austria, were eligible for inclusion.

Exclusion criteria included chronic analgesic therapy, opioid intake within 12 h prior to surgery, history of drug or alcohol abuse, contraindications to metamizole, inability to understand pain scoring, ophthalmologic disease, medication affecting pupillary function (beta-antagonists, anti-emetics, atropine, phenylephrine), implanted electronic devices, neurological or psychiatric disorders, need for rapid sequence induction, or anticipated postoperative ventilation.

### 2.2. Anesthesia Protocol

Anesthesia was induced and maintained with propofol (2–5 mg∙kg^−1^ for induction, 2–10 mg∙kg^−1^∙h^−1^ for maintenance) and remifentanil (0.1–0.5 µg∙kg^−1^∙min^−1^), with rocuronium (0.6 mg∙kg^−1^) used to facilitate intubation. Mechanical ventilation with an oxygen-air mixture was adjusted to maintain normocapnia (end-tidal CO_2_ 30–40 mmHg). Both groups were managed using the same anesthetic regimen. All patients received 500 mg of intravenous metamizole as part of the standardized multimodal analgesic regimen, administered at least 30 min before the end of surgery.

### 2.3. Randomization

Patients were randomized 1:1 either to PPI-guided opioid therapy or standard care. Randomization was performed using computer-generated (randomizer software, URL https://www.meduniwien.ac.at/randomizer, (accessed on 25 April 2020), Randomization Service for Multicenter Clinical Trials; Randomizer Version 2.1.0; Medical University Vienna, Austria) allocation with block randomization stratified by age, sex, and surgery type (ear/nose vs. throat).

### 2.4. Intervention

In the PPI-guided group, PPI (range 1–9) was measured using an infrared pupilometer (set to PPI mode) 10 min after opioid cessation. A PPI > 3, indicating a mild or more severe nociception level, triggered piritramide administration (initial 0.1 mg∙kg^−1^, followed by 0.05 mg∙kg^−1^ for subsequent boluses). Measurement and dosing were repeated every 3 min until PPI ≤ 3. Non-responders (unchanged or increasing PPI) did not receive any further opioids.

In the non-PPI-guided group (standard care), a single blinded pupillometry measurement was performed 10 min after cessation of opioids. Opioid administration followed standard clinical practice with 3 mg piritramide boluses for NRS > 3.

### 2.5. Endpoints

The primary endpoint was total opioid consumption during the first 2 postoperative hours in the postoperative anesthesia care unit (PACU). Secondary endpoints included mean NRS pain scores during the first two postoperative hours. Exploratory endpoints were time to first opioid administration in the PACU and total intraoperative remifentanil dose.

### 2.6. Measurements

Opioid consumption and NRS pain levels were recorded every 30 min during the first 2 postoperative hours. Additional data collected included NRS pain level upon arrival at the PACU, time to first opioid administration in the PACU, total intraoperative remifentanil consumption, depth of anesthesia (assessed by electroencephalography; Narcotrend, Monitor Technik, 30625 Hannover, Germany), and side effects such as postoperative nausea and vomiting, respiratory depression, desaturation, and bradypnea.

PPI was measured bilaterally using an automated infrared portable pupilometer (AlgiScan, IDMed, 13013 Marseille, France) with standardized electrical stimuli (10–60 mA, 1 s duration, pulse width 200 μs). Stimulation was stopped at ≥13% pupillary dilation. The PPI score was automatically calculated by the pupillometer using proprietary manufacturer-defined software. The lower the PPI score, the greater the opioid receptor occupancy, which suggests lower nociception.

Pupillometry was performed on both eyes with a rubber cup placed in the orbit to ensure optimal device position, pupil-camera distance, and environmental darkness. The contralateral eye was closed to minimize consensual light response. Since physiological anisocoria (0.4 mm) occurs in 10–20% of the population, the mean PPI of both eyes was calculated for further analyses [19,20]. Pupillometry was performed immediately before emergence from general anesthesia, as the procedure is not suitable for awake patients.

### 2.7. Statistical Analysis

Demographic and morphometric characteristics were summarized as median and first and third quartiles for continuous variables and frequencies and percentages for categorical variables.

Primary and secondary endpoints were compared between both study groups using univariable linear regression as well as multivariable ANCOVA models. These models contain the treatment group as the main predictor and adjust for factors such as remifentanil and lidocaine dose, weight, gender, and type of surgery (nose vs. no nose) and the interaction between treatment group and surgery type (nasal vs. no nasal), lidocaine total (yes/no) group, and epinephrine total (yes/no) group. Additionally, we performed bivariate tests for individual measurement time points of opioid consumption and NRS scores. Exploratory analyses were performed with the chi-squared test, Fisher’s exact test, and Wilcoxon rank-sum tests.

The sample size calculation was based on a two-sample *t*-test approach, assuming a minimal relevant difference in opioid consumption of 3 mg (equivalent to one bolus), with an estimated standard deviation of 4.13 mg. This standard deviation corresponds to a slightly skewed log-normal distribution with a median of 9 mg and a 90th percentile of 15 mg, derived from preliminary data. To achieve 80% power at a two-sided significance level of 0.05, a sample size of 31 patients per group (total *N* = 62) was required. All analyses were conducted using R studio, version 2024.04.2 (Posit team, 2024), with a significance threshold set at 0.05.

## 3. Results

**Figure 1:**
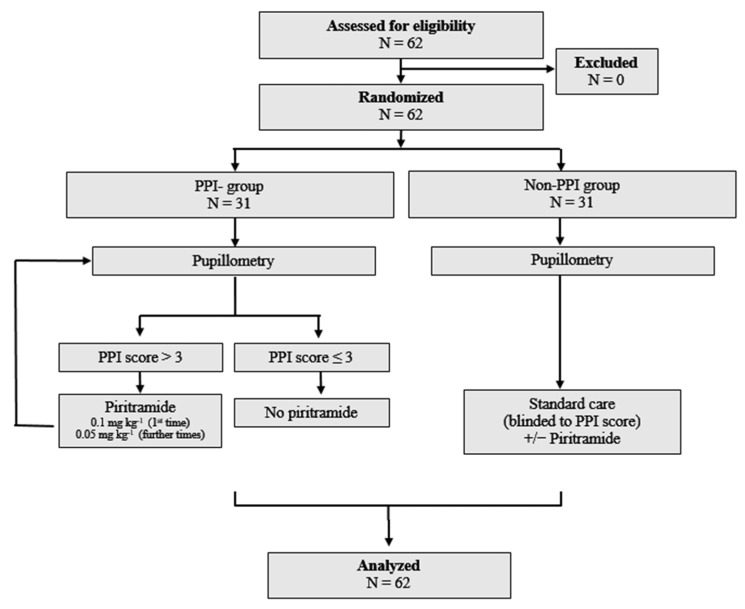
Flow chart.

A total of 62 patients were enrolled in this study, with 31 patients randomized to the PPI-guided group and 31 to the standard care group (non-PPI-guided group). (Figure 1). Demographic characteristics, including age, sex, body mass index, ASA physical status, type of surgery, and intraoperative anesthetic variables, are summarized in Table 1.

Intraoperative anesthetic management was similar, except for higher piritramide use in the PPI-guided group, which was consistent with the protocolized opioid administration. Median intraoperative remifentanil infusion rates were similar among both groups.

### 3.1. Primary Endpoint: Opioid Consumption

**Figure 2:**
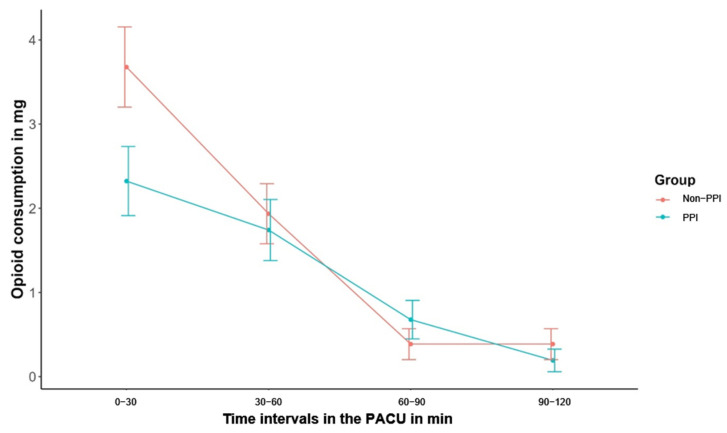
Opioid consumption (piritramide, mg) in 30-min intervals during the first 2 postoperative hours. Data are presented with means ± standard errors. Total opioid consumption during the first 2 postoperative hours did not differ between groups (*p* = 0.16). Opioid consumption was significantly lower in the PPI group during the first 30 min (median 3 [0; 3] mg vs. 3 [3; 6] mg, *p* = 0.03). No significant differences were observed between groups at subsequent intervals. PPI, pupillary pain index-guided pain therapy; non-PPI, non-pupillary pain index-guided pain therapy-standard care group; PACU, Post Anesthesia Care Unit.

Total opioid consumption during the first postoperative 2 h did not differ significantly between groups (mean difference: −1.55, 95%CI [−3.72, 0.62] mg, *p* = 0.16; adjusted mean difference: −1.68 [−3.85; 0.48] mg, *p* = 0.12; Table 2). However, during the first 30 min, opioid consumption was significantly lower in the PPI-guided group compared to standard care (3 [0; 3] mg versus 3 [3; 6] mg, *p* = 0.03 (Figure 2, Table 3)). No significant differences were observed at subsequent time intervals. Overall, 76% (n = 47) of all patients required at least one opioid dose during the PACU stay. In the standard care group, 84% (26 of 31 patients) received opioids compared to 68% (21 of 31 patients) in the PPI group.

Overall opioid consumption (OR and PACU; piritramide, mg) showed a significant difference between the two study groups in the univariable analysis (mean difference 3.0 mg, 95% CI [0.1, 5.97], *p* = 0.04; Table 2), favoring the PPI group. After adjusting for remifentanil dosage (ug^−1^kg^−1^min^−1^), weight (kg), type of operation (nose/non-nose), and gender, the estimated difference was 2.8 mg (95% CI [−0.08, 5.74], *p* = 0.06). The time of first opioid consumption in recovery did not differ between groups (Table 3).

### 3.2. Secondary Endpoint: Postoperative Pain

**Figure 3:**
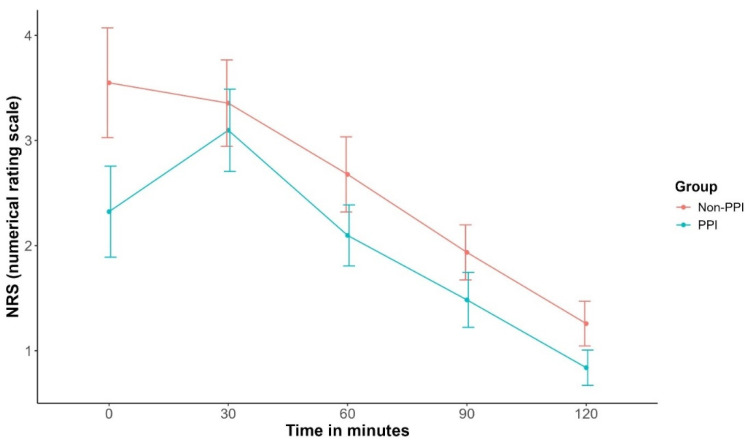
Numeric rating scale pain values (NRS) in 30-min intervals over the first 2 postoperative hours. Data are presented with means ± standard errors. Total pain scores within the first 2 postoperative hours showed no statistically significant difference between groups (mean difference −0.59, 95%CI [−1.24, 0.06], *p* = 0.08). The median (IQR) pain score at PACU arrival was 2 (0; 4) in the PPI group and 4 (0; 5) in the non-PPI group. PPI, pupillary pain index-guided pain therapy; PACU, Post Anesthesia Care Unit.

The average pain over the first postoperative 2 h did not differ significantly between groups (mean difference −0.59, 95% CI [−1.24, 0.06], *p* = 0.08). (Table 3) The median (IQR) NRS pain scores on PACU arrival were lower in the PPI group (median NRS 2 vs. 4), although not statistically significant. Subsequent pain scores, measured at 30-min intervals over the following two hours, were comparable between groups (Figure 3; Table 3).

### 3.3. Exploratory Endpoints

**Figure 4:**
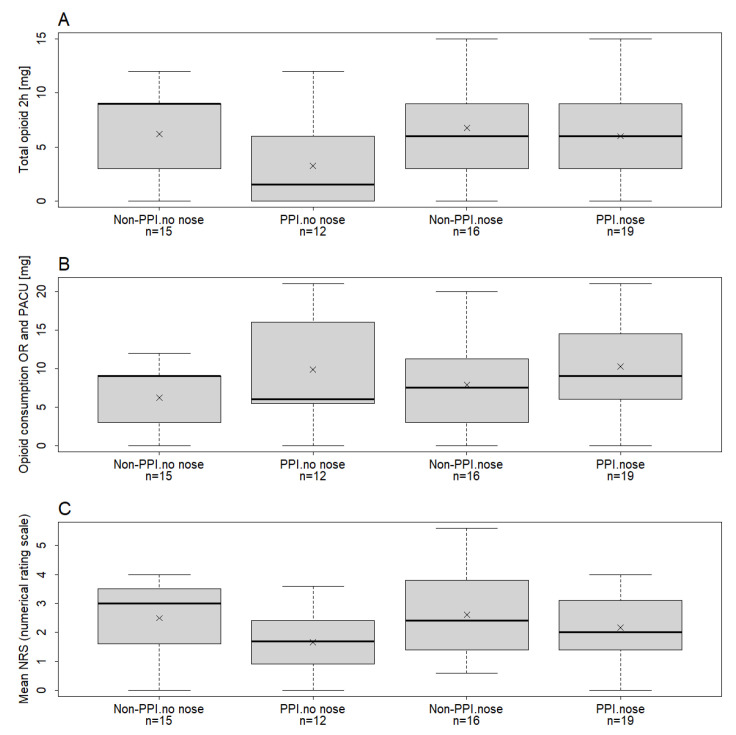
Boxplots illustrating total opioid consumption (mg) during the first 2-h PACU stay (Panel **A**), cumulative opioid consumption (OR and PACU) (Panel **B**), and mean Numerical Rating Scale (NRS) (Panel **C**). Boxplots are stratified by treatment group (PPI vs. non-PPI) and surgery type (nose vs. no-nose). The four subgroups are as follows: PPI–nose (nasal surgery in the PPI group), PPI–no-nose (ear and throat surgery in the PPI group), non-PPI–nose (nasal surgery in the standard care group), and non-PPI–no-nose (ear and throat surgery in the standard care group). Each category is displayed once to avoid duplication. Crosses indicate the group-specific sample mean. (Panel **A**) Distribution of total opioid consumption (mg) during the first 2 postoperative hours in the PACU. Boxplots depict median values, interquartile ranges, and outliers for each study group. (Panel **B**) Cumulative intraoperative and postoperative opioid requirements, demonstrating overall perioperative analgesic demand. (Panel **C**) Mean pain intensity scores (NRS 0–10), with higher values indicating greater pain severity (0 = no pain, 10 = worst imaginable pain). Horizontal markers (crosses) represent arithmetic means for each cohort. Non-parametric statistical methods were employed due to non-normal distributions of opioid consumption and pain scores.

Figure 4 summarizes subgroup analyses stratified by surgery type. In the standard-care group, nasal versus non-nasal (ear, throat) surgery did not influence postoperative opioid consumption (mean difference 1.10, 95% CI (−2.08, 4.28), *p* = 0.49). In contrast, within the PPI-guided group, patients undergoing nasal surgery tended to require more opioids (mean difference 3.04 mg; 95% CI −0.24 to 6.32; *p* = 0.069), although this did not reach statistical significance.

The final observed intraoperative PPI score correlated with the initial NRS score at PACU arrival (Spearman r = 0.30, *p* = 0.02) but did not correlate with total opioid consumption (Spearman r = 0.05, *p* = 0.69). The first postoperative PPI score was similar between both study groups (*p* = 0.55; Table 2). Intraoperative use of lidocaine, epinephrine, and remifentanil consumption did not significantly affect the first postoperative PPI score.

Total intraoperative remifentanil dose did not correlate with total postoperative opioid consumption (r = −0.07, *p* = 0.57) and postoperative NRS pain scores (r = 0.02, *p* = 0.86). The total intraoperative remifentanil dose was similar across types of surgery (median (IQR) nose: 0.28 (0.24; 0.3) vs. non-nose: 0.29 (0.25; 0.34); *p* = 0.13).

Neither intraoperative remifentanil administration nor the use of topical or infiltrative lidocaine or epinephrine was associated with postoperative opioid consumption (*p* = 0.24; *p* = 0.40) or pain (*p* = 0.61; *p* = 0.844).

No opioid-related adverse events, including postoperative nausea and vomiting, bradycardia, hypotension, respiratory depression, desaturation, or bradypnea, were observed in either group during the PACU stay. Therefore, statistical comparison was not applicable.

Gender-related differences were limited. Female patients had higher PPI values (median (IQR) female: 5 (2.75; 7) vs. male: 2.5 (1.5; 5.75); *p* = 0.038) before emergence. Pain at PACU arrival and opioid consumption during the first postoperative 30 min were higher in women, although not statistically significant (median (IQR) female: 3 (1.5; 6) vs. male: 3 (0; 3); *p* = 0.33). First opioid request, overall opioid consumption, and NRS pain score during the first 2 postoperative hours were comparable.

## 4. Discussion

In this randomized controlled trial, PPI-guided opioid administration did not reduce total opioid consumption or mean postoperative pain scores during the first 2 h after ENT surgery. These findings contrast with previous studies that demonstrated intraoperative PPI-guided remifentanil administration could reduce postoperative pain and analgesic requirements up to 12 h postoperatively [15,16,17,18]. However, consistent with alternate earlier reports, we observed that PPI guidance reduced opioid consumption during the first 30 min postoperatively and lowered median pain levels at PACU arrival. These results suggest that the benefit of PPI-guided opioid administration may be limited to early postoperative pain control in patients recovering from ENT surgery. The observed early opioid-sparing effect may reduce the immediate need for opioid administration, potentially improving patient comfort upon PACU arrival and decreasing the risk of opioid-related side effects, such as nausea, vomiting, sedation, and respiratory depression, all of which can negatively impact a patient’s overall well-being and recovery [21,22,23,24]. Notably, no opioid-related adverse events such as postoperative nausea and vomiting or respiratory depression were observed in either group during the PACU stay.

While no significant differences in total opioid consumption were found after the first 2 h, the early reduction in opioid requirement during the first 30 min is clinically relevant. This period is characterized by heightened vulnerability to opioid-induced side effects. Even a modest opioid-sparing effect at this timepoint may thus enhance patient comfort, reduce the need for rescue anti-emetics, support a smoother early recovery, and ultimately increase patient safety. Although our study was not powered to detect differences in adverse events, this observation warrants further investigation.

Postoperative pain following ENT surgery can vary widely among patients [25,26]. Several studies have shown that surgeries in the neck area (pharynx and larynx) are among the most painful ENT surgeries, while ear surgeries tend to cause less significant pain [26,27]. In our study, pain scores upon PACU arrival were lower in the PPI group (NRS 2) compared to the standard care group (NRS 4). Notably, 23 patients did not require any opioids at the end of surgery, anticipating minimal postoperative pain. These patients had NRS scores ≤ 3 upon PACU arrival and required an average of 3.91 mg piritramide, with a mean NRS of 1.63 during the first 2 postoperative hours. This suggests that many patients underwent procedures with low opioid requirements postoperatively, which may have limited the ability to detect significant differences between the groups.

Importantly, the overall pain intensity in our cohort was low, with median NRS values of 2–4 upon PACU arrival, and nearly one-third of patients required no intraoperative opioid administration. When postoperative nociceptive burden is minimal, additional titration strategies—such as PPI guidance—may not result in measurable reductions in opioid administration or pain scores. The limited analgesic requirements in many patients, therefore, need to be considered when interpreting the neutral primary outcome. Given the substantial variability in postoperative pain across ENT procedures, it is possible that PPI guidance may have greater clinical impact in more painful surgeries. Future trials focusing on high-intensity nociceptive interventions could help clarify whether the benefits of pupillometry become more pronounced under conditions of greater analgesic demand.

Consistent with previous studies, the pupil dilation reflex (PDR) reflects the balance between nociception and antinociception and is dose-dependently suppressed by opioids [12,28,29,30]. PDR has been shown to correlate with opioid requirements during controlled pressure applied to a surgical wound [1] and with verbal pain assessments [31,32,33]. Despite these findings, the results of our trial indicate that PPI-guided opioid analgesia does not reduce postoperative opioid consumption to a clinically meaningful extent. However, we did observe that the most recent PPI score in the OR significantly correlated with the first NRS score upon arrival in the PACU, suggesting that PPI may serve as a predictor of pain in the early postoperative period. Pain and nociception were clearly distinguished throughout the study. Nociception during anesthesia was quantified using the PPI, whereas postoperative pain was assessed in awake patients using the NRS.

Previous studies have demonstrated that PPI-guided remifentanil administration reduces postoperative pain and opioid consumption [15,16,17,18]. However, these studies focused on using PPI to guide the continuous administration of a short-acting opioid during anesthesia, where the effects of the opioid typically diminish postoperatively. Our sub-analysis showed that the intraoperative administration of remifentanil was similar across treatment groups and surgery types and did not correlate with postoperative pain levels or opioid consumption.

Several anesthetics are known to influence pupillary reflexes [9,10,11,12,13,34,35]. At analgesic doses, opioids reduce the amplitude of PDR in response to standardized noxious stimuli, providing a practical method for assessing analgesic effectiveness and titrating opioid dosages. However, in endoscopic sinus surgery, nasal decongestants such as a solution of lidocaine hydrochloride and phenylephrine hydrochloride are often used to minimize blood loss and enhance visibility. These substances may enter the eye through the nasolacrimal duct, potentially causing mydriasis or alterations in pupillary reflexes [36,37]. This could influence PPI measurements during nasal surgery. To account for this, we conducted a sub-analysis on the administration of lidocaine and epinephrine, which showed no significant impact on outcomes. Similarly, analysis across all types of ENT surgeries (nose vs. non-nose (ear, throat)) did not reveal any discernible impact on PPI scores, opioid consumption, or pain in either treatment group.

Regarding gender-specific effects, no significant differences in the primary outcome were observed. Although women had higher first PPI scores recorded in the operating room, and their first opioid administration in the PACU occurred earlier, these differences did not extend to other relevant outcomes. Women generally have lower pain thresholds and less experimental pain tolerance, which may have contributed to their higher levels of pain after surgery [38,39,40,41,42,43,44,45,46,47,48,49,50]. These findings align with those observed in the ENT surgery population by Summer et al. [26]. Human studies have demonstrated that women exhibit higher pain intensity and require up to 30% more morphine on a per-weight basis compared to men to achieve similar reductions in pain intensity [40,51]. However, other studies suggest that gender may not significantly affect postoperative pain experiences [52].

This study has several limitations. First, the sample size was insufficient for robust subgroup analysis, limiting the statistical power to detect differences within subgroups (e.g., surgery types). The most common surgery type in our study required only low intraoperative opioid doses, suggesting low postoperative pain levels and opioid requirements, which may have masked any clinically relevant difference. Second, no second PPI measurement was conducted in the non-PPI group, which could have provided further insights into postoperative pain management. Third, the PPI score is automatically generated based on graded nociceptive stimulation. The short intervals between stimulation levels (10–60 mA) may not allow sufficient time for full recovery of the pupillary reflex. A tailored stimulation intensity, aligned with the extent of the surgical intervention, may therefore be more effective in capturing an accurate reflex response. Larger trials with varying stimulation intensities are needed to refine this approach. A further limitation is that PPI monitoring was performed only at the end of surgery. Continuous intraoperative pupillometry was not feasible because the procedures involved ENT surgery, where the surgical field and patient positioning impede direct access to the eyes. As a result, remifentanil administration during earlier intraoperative phases followed standard clinical practice. Lastly, the absence of a second PPI measurement in the control group, though intentionally omitted to avoid contamination of the standard care pathway, limits mechanistic insight into the temporal relationship between intraoperative PPI dynamics and postoperative pain outcomes. As a result, comparisons of PPI trajectories between groups were not possible.

## 5. Conclusions

In conclusion, PPI-guided opioid analgesia did not reduce postoperative opioid consumption or pain during the first postoperative 2 h following ENT surgery. However, PPI guidance did reduce immediate postoperative opioid use and pain, suggesting at least some minimal benefit in the early recovery phase after surgery. Notably, the last intraoperatively measured PPI index correlated with initial pain levels in the PACU, highlighting its potential as a predictor of early postoperative pain.

## Figures and Tables

**Table 1:** Baseline and perioperative patient characteristics.

	All Patients (N = 62)	PPI-Guided (N = 31)	Non-PPI-Guided (N = 31)	*p*
Age, years	33 (23, 46)	33 (23, 48)	32 (24, 45)	0.98
Female	31 (50)	16 (52)	15 (48)	1
Height, cm	170 (165, 179)	170 (165, 176)	170 (164, 180)	0.73
Weight, kg	74 (65, 83)	74 (66, 83)	74 (65, 87)	0.89
BMI, kg m^−2^	24.7 (22.2, 27.4)	24.6 (21.5, 27.3)	24.9 (22.6, 27.3)	0.49
ASA status				0.73
1	52 (84)	25 (81)	27 (87)	
2	10 (16)	6 (19)	4 (13)	
Type of surgery				0.61
Nasal surgery	35 (56)	19 (61)	16 (52)	
*Endoscopic Sinus Surgery*	10 (16)	3 (10)	7 (23)	
*Septorhinoplasty*	25 (40)	16 (52)	9 (29)	
Non-nasal surgery	27 (44)	12 (39)	15 (48)	
Non-nasal surgery—Ear	12 (19)	6 (19)	6 (19)	
*Tympanoplasty*	7 (11)	3 (10)	4 (13)	
*Cochlear Implantation*	2 (3)	1 (3)	1 (3)	
*Stapedoplasty*	1 (2)	1 (3)	0 (0)	
*Mastoidectomy*	2 (3)	1 (3)	1(3)	
Non-nasal surgery—Throat	15 (24)	6 (19)	9 (29)	
*Tonsillectomy*	5 (8)	1 (3)	4 (13)	
*Cervical Cyst Excision*	2 (3)	1 (3)	1 (3)	
*Hypopharyngeal Surgery*	2 (3)	1 (3)	1 (3)	
*Parotid Surgery*	4 (7)	2 (7)	2 (7)	
*Microlaryngoscopy*	1 (2)	0 (0)	1 (3)	
*Cervical Lymphadenectomy*	1 (2)	1 (3)	0 (0)	
*Intraoperative*				
Duration of anesthesia, min	121 (78, 178)	137 (85, 188)	121 (78, 162)	0.34
Remifentanil, ug ^−1^ kg^−1^ min^−1^	0.28 (0.24, 0.33)	0.26 (0.24, 0.31)	0.28 (0.24, 0.33)	0.45
Piritramide, OR, mg	0 (0, 5)	5 (0, 8)	0 (0, 0)	<0.001
PPI at baseline	4.5 (2, 6)	4.5 (2, 5.75)	4 (2, 6.5)	0.467
Infiltration—lidocaine, epinephrine, mL	2 (0, 6)	2 (0, 14)	2 (0, 5)	0.26
Nasal packing with lidocaine, rhinon	33 (53)	18 (58)	15 (48)	0.61
Nasal packing with epinephrine	19 (31)	13 (42)	6 (19)	0.09

Data are presented with median (Q1; Q3) or n (%). Differences were assessed with the Wilcoxon test for continuous variables and with the chi-square or Fisher’s exact test for categorical variables. For continuous variables, values are presented as median (Q1; Q3), and for categorical variables as counts (percent). Differences in distribution between the groups for continuous variables were analyzed with the Wilcoxon rank sum test, while categorical variables were compared using the chi-square or Fisher’s exact test.

**Table 2:** Comparison of primary and secondary study endpoints between treatment groups.

	Summary Statistics Mean (SD)		Univariable Analysis			Multivariable Analysis		
Endpoints	PPI-Guided	Non-PPI-Guided	Mean Difference	95% CI	*p*	Mean Difference	95% CI	*p*
Total opioid consumption during the first 2 h in PACU [piritramide, mg]	4.94 (4.28)	6.48 (4.25)	−1.55	[−3.72; 0.62]	0.16	−1.68	[−3.85; 0.48]	0.12
Mean NRS during the first 2 postoperative hours in PACU	1.97 (1.12)	2.55 (1.42)	−0.59	[−1.24; 0.06]	0.08	−0.553	[−1.22; 0.12]	0.10

Multivariable linear models were adjusted for remifentanil dose (ug∙kg^−1^∙min^−1^), weight (kg), type of surgery (nose/non-nose), and sex. Models with Numerical Rating Scale (NRS) as the outcome variable were not adjusted for the type of surgery. OR, operating room; PPI, pupillary pain index; PACU, Post Anesthesia Care Unit.

**Table 3:** Opioid consumption and pain scores during the first 2 postoperative hours.

	All (N = 62)	PPI-Guided (N = 31)	Non-PPI-Guided (N = 31)	*p*
**Opioid consumption, mg**				
0–30 min	3 (0, 3)	3 (0, 3)	3 (3, 6)	0.03
30–60 min	3 (0, 3)	0 (0, 3)	3 (0, 3)	0.67
60–90 min	0 (0, 0)	0 (0, 0)	0 (0, 0)	0.33
90–120 min	0 (0, 0)	0 (0, 0)	0 (0, 0)	0.40
**Time to first opioid consumption in PACU, min**	5 (0, 15)	5 (0, 19)	5 (1.5, 15)	0.59
**Pain score during PACU at**				
0 min-arrival at PACU	3 (0, 5)	2 (0, 4)	4 (0, 5)	0.08
30 min	3 (2, 5)	3 (1.5, 4)	3 (2, 5)	0.60
60 min	2 (1, 3)	2 (1, 3)	3 (1, 3.5)	0.23
90 min	2 (0, 3)	1 (0, 3)	2 (1, 3)	0.23
120 min	1 (0, 2)	1(0, 1)	1 (0, 2)	0.17

Data are presented as median (Q1; Q3). Statistical comparisons were performed with the Wilcoxon rank sum test, PPI, pupillary pain index; OR, operating room; PACU, Post Anesthesia Care Unit; NRS, numeric rating scale (range 0–10).

## Data Availability

The data presented in this study are available on reasonable request from the corresponding author.

## Abbreviations

The following abbreviations are used in this manuscript:

ANI	Analgesia Nociception Index
ENT	Ear Nose Throat
NoL	Nociception Level Index
PACU	Post Anesthesia Care Unit
PDR	Pupillary dilation reflex
PPI	Pupillary pain index
SPI	Surgical Pleth Index

## References

[1] Aissou M., Snauwaert A., Dupuis C., Atchabahian A., Aubrun F., Beaussier M. (2012). Objective assessment of the immediate postoperative analgesia using pupillary reflex measurement: A prospective and observational study. Anesthesiology.

[2] Pogatzki-Zahn E.M., Segelcke D., Schug S.A. (2017). Postoperative pain-from mechanisms to treatment. Pain Rep..

[3] Ledowski T. (2019). Objective monitoring of nociception: A review of current commercial solutions. Br. J. Anaesth..

[4] Hohenschurz-Schmidt D.J., Calcagnini G., Dipasquale O., Jackson J.B., Medina S., O’Daly O., O’Muircheartaigh J., de Lara Rubio A., Williams S.C.R., McMahon S.B. (2020). Linking Pain Sensation to the Autonomic Nervous System: The Role of the Anterior Cingulate and Periaqueductal Gray Resting-State Networks. Front. Neurosci..

[5] Ben-Israel N., Kliger M., Zuckerman G., Katz Y., Edry R. (2013). Monitoring the nociception level: A multi-parameter approach. J. Clin. Monit. Comput..

[6] Martini C.H., Boon M., Broens S.J., Hekkelman E.F., Oudhoff L.A., Buddeke A.W., Dahan A. (2015). Ability of the nociception level, a multiparameter composite of autonomic signals, to detect noxious stimuli during propofol-remifentanil anesthesia. Anesthesiology.

[7] Meijer F.S., Martini C.H., Broens S., Boon M., Niesters M., Aarts L., Olofsen E., van Velzen M., Dahan A. (2019). Nociception-guided versus Standard Care during Remifentanil-Propofol Anesthesia: A Randomized Controlled Trial. Anesthesiology.

[8] Chapman C.R., Oka S., Bradshaw D.H., Jacobson R.C., Donaldson G.W. (1999). Phasic pupil dilation response to noxious stimulation in normal volunteers: Relationship to brain evoked potentials and pain report. Psychophysiology.

[9] Larson M.D., Sessler D.I., Washington D.E., Merrifield B.R., Hynson J.A., McGuire J. (1993). Pupillary response to noxious stimulation during isoflurane and propofol anesthesia. Anesth. Analg..

[10] Knaggs R.D., Crighton I.M., Cobby T.F., Fletcher A.J., Hobbs G.J. (2004). The pupillary effects of intravenous morphine, codeine, and tramadol in volunteers. Anesth. Analg..

[11] Larson M.D. (2008). Mechanism of opioid-induced pupillary effects. Clin. Neurophysiol..

[12] Larson M.D., Kurz A., Sessler D.I., Dechert M., Bjorksten A.R., Tayefeh F. (1997). Alfentanil blocks reflex pupillary dilation in response to noxious stimulation but does not diminish the light reflex. Anesthesiology.

[13] Lee H.K., Wang S.C. (1975). Mechanism of morphine-induced miosis in the dog. J. Pharmacol. Exp. Ther..

[14] Isnardon S., Vinclair M., Genty C., Hebrard A., Albaladejo P., Payen J.F. (2013). Pupillometry to detect pain response during general anaesthesia following unilateral popliteal sciatic nerve block: A prospective, observational study. Eur. J. Anaesthesiol..

[15] Sabourdin N., Barrois J., Louvet N., Rigouzzo A., Guye M.L., Dadure C., Constant I. (2017). Pupillometry-guided Intraoperative Remifentanil Administration versus Standard Practice Influences Opioid Use: A Randomized Study. Anesthesiology.

[16] Abad Torrent A., Rodriguez Bustamante V., Carrasco Fons N., Roca Tutusaus F.J., Blanco Vargas D., Gonzalez Garcia C. (2016). The use of pupillometry as monitoring of intraoperative analgesia in the consumption of analgesics during the first 12 hours after surgery. Rev. Esp. De Anestesiol. Y Reanim..

[17] Wildemeersch D., Peeters N., Saldien V., Vercauteren M., Hans G. (2018). Pain assessment by pupil dilation reflex in response to noxious stimulation in anaesthetized adults. Acta Anaesthesiol. Scand..

[18] Oka S., Chapman C.R., Kim B., Nakajima I., Shimizu O., Oi Y. (2007). Pupil dilation response to noxious stimulation: Effect of varying nitrous oxide concentration. Clin. Neurophysiol..

[19] Lam B.L., Thompson H.S., Corbett J.J. (1987). The prevalence of simple anisocoria. Am. J. Ophthalmol..

[20] Jindal M., Sharma N., Parekh N. (2009). Intraoperative dilated pupil during nasal polypectomy. Eur. Arch. Oto-Rhino-Laryngol..

[21] Rogers E., Mehta S., Shengelia R., Reid M.C. (2013). Four Strategies for Managing Opioid-Induced Side Effects in Older Adults. Clin. Geriatr..

[22] Angst M.S., Clark J.D. (2006). Opioid-induced hyperalgesia: A qualitative systematic review. Anesthesiology.

[23] Hyland S.J., Brockhaus K.K., Vincent W.R., Spence N.Z., Lucki M.M., Howkins M.J., Cleary R.K. (2021). Perioperative Pain Management and Opioid Stewardship: A Practical Guide. Healthcare.

[24] Kehlet H., Wilmore D.W. (2002). Multimodal strategies to improve surgical outcome. Am. J. Surg..

[25] Guntinas-Lichius O., Volk G.F., Zaslansky R., Meissner W. (2014). The first postoperative day: Prospective evaluation of pain in adult otorhinolaryngologic surgery. Clin. J. Pain.

[26] Sommer M., Geurts J.W., Stessel B., Kessels A.G., Peters M.L., Patijn J., van Kleef M., Kremer B., Marcus M.A. (2009). Prevalence and predictors of postoperative pain after ear, nose, and throat surgery. Arch. Otolaryngol.—Head Neck Surg..

[27] Wittekindt D., Wittekindt C., Schneider G., Meissner W., Guntinas-Lichius O. (2012). Postoperative pain assessment after septorhinoplasty. Eur. Arch. Oto-Rhino-Laryngol..

[28] Barvais L., Engelman E., Eba J.M., Coussaert E., Cantraine F., Kenny G.N. (2003). Effect site concentrations of remifentanil and pupil response to noxious stimulation. Br. J. Anaesth..

[29] Vlaenderen D.V., Hans G., Saldien V., Wildemeersch D. (2022). Pupillary reflex dilation and pain index evaluation during general anesthesia using sufentanil: A double-blind randomized controlled trial. Pain Manag..

[30] Wildemeersch D., Baeten M., Peeters N., Saldien V., Vercauteren M., Hans G. (2018). Pupillary dilation reflex and pupillary pain index evaluation during general anaesthesia: A pilot study. Rom. J. Anaesth. Intensive Care.

[31] Connelly M.A., Brown J.T., Kearns G.L., Anderson R.A., St Peter S.D., Neville K.A. (2014). Pupillometry: A non-invasive technique for pain assessment in paediatric patients. Arch. Dis. Child..

[32] Ly-Liu D., Reinoso-Barbero F. (2015). Immediate postoperative pain can also be predicted by pupillary pain index in children. Br. J. Anaesth..

[33] Kornilov E., Gehlen L., Yacobi D., Soehle M., Kowark A., Thudium M. (2023). Pupillary Pain Index Predicts Postoperative Pain but Not the Effect of Peripheral Regional Anaesthesia in Patients Undergoing Total Hip or Total Knee Arthroplasty: An Observational Study. Medicina.

[34] Larson M.D., Berry P.D., May J., Bjorksten A., Sessler D.I. (2004). Latency of pupillary reflex dilation during general anesthesia. J. Appl. Physiol..

[35] Leslie K., Sessler D.I., Smith W.D., Larson M.D., Ozaki M., Blanchard D., Crankshaw D.P. (1996). Prediction of movement during propofol/nitrous oxide anesthesia. Performance of concentration, electroencephalographic, pupillary, and hemodynamic indicators. Anesthesiology.

[36] D’Souza M.G., Hadzic A., Wider T. (2000). Unilateral mydriasis after nasal reconstruction surgery. Can. J. Anaesth..

[37] Rubin M.M., Sadoff R.S., Cozzi G.M. (1990). Postoperative unilateral mydriasis due to phenylephrine: A case report. J. Oral Maxillofac. Surg..

[38] Riley J.L., Robinson M.E., Wise E.A., Price D. (1999). A meta-analytic review of pain perception across the menstrual cycle. Pain.

[39] Kest B., Sarton E., Dahan A. (2000). Gender differences in opioid-mediated analgesia: Animal and human studies. Anesthesiology.

[40] Cepeda M.S., Carr D.B. (2003). Women experience more pain and require more morphine than men to achieve a similar degree of analgesia. Anesth. Analg..

[41] Chia Y.Y., Chow L.H., Hung C.C., Liu K., Ger L.P., Wang P.N. (2002). Gender and pain upon movement are associated with the requirements for postoperative patient-controlled iv analgesia: A prospective survey of 2298 Chinese patients. Can. J. Anaesth..

[42] De Cosmo G., Congedo E., Lai C., Primieri P., Dottarelli A., Aceto P. (2008). Preoperative psychologic and demographic predictors of pain perception and tramadol consumption using intravenous patient-controlled analgesia. Clin. J. Pain.

[43] Gagliese L., Gauthier L.R., Macpherson A.K., Jovellanos M., Chan V.W. (2008). Correlates of postoperative pain and intravenous patient-controlled analgesia use in younger and older surgical patients. Pain Med..

[44] Lau H., Patil N.G. (2004). Acute pain after endoscopic totally extraperitoneal (TEP) inguinal hernioplasty: Multivariate analysis of predictive factors. Surg. Endosc..

[45] Caumo W., Schmidt A.P., Schneider C.N., Bergmann J., Iwamoto C.W., Adamatti L.C., Bandeira D., Ferreira M.B. (2002). Preoperative predictors of moderate to intense acute postoperative pain in patients undergoing abdominal surgery. Acta Anaesthesiol. Scand..

[46] Kalkman J.C., Visser K., Moen J., Bonsel J.G., Grobbee E.D., Moons M.K.G. (2003). Preoperative prediction of severe postoperative pain. Pain.

[47] Mamie C., Bernstein M., Morabia A., Klopfenstein C.E., Sloutskis D., Forster A. (2004). Are there reliable predictors of postoperative pain?. Acta Anaesthesiol. Scand..

[48] Unruh A.M. (1996). Gender variations in clinical pain experience. Pain.

[49] Cepeda M.S., Africano J.M., Manrique A.M., Fragoso W., Carr D.B. (2002). The combination of low dose of naloxone and morphine in PCA does not decrease opioid requirements in the postoperative period. Pain.

[50] Barsky A.J., Peekna H.M., Borus J.F. (2001). Somatic symptom reporting in women and men. J. Gen. Intern. Med..

[51] Fillingim R.B., King C.D., Ribeiro-Dasilva M.C., Rahim-Williams B., Riley J.L. (2009). Sex, gender, and pain: A review of recent clinical and experimental findings. J. Pain.

[52] Ip H.Y., Abrishami A., Peng P.W., Wong J., Chung F. (2009). Predictors of postoperative pain and analgesic consumption: A qualitative systematic review. Anesthesiology.

